# Influence of Minimum-Duration Processing Settings on activPAL-Derived Sit-to-Stand Transitions and Sedentary Behavior Metrics in Free-Living Chronic Stroke Survivors: An Exploratory Pilot Study

**DOI:** 10.7759/cureus.113847

**Published:** 2026-08-03

**Authors:** Mitsutaka Shibuya, Kazuhiro Harada, Haruo Uguisu, Kenichi Hirashima

**Affiliations:** 1 Department of Physical Therapy, Faculty of Health and Welfare, Tokushima Bunri University, Tokushima, JPN; 2 Graduate School of Health Science Studies, Kibi International University, Okayama, JPN

**Keywords:** activpal, chronic stroke survivors, minimum non-upright period, minimum upright period, sedentary behavior, sit-to-stand transitions

## Abstract

Background and objective

The activPAL monitor (PAL Technologies, Glasgow, UK) is widely used to assess posture, stepping activity, sit-to-stand transitions, and sedentary behavior. However, estimates derived from the same activPAL recording may vary according to the minimum-duration settings applied during data processing. Such processing-related differences may affect the interpretation of daily sit-to-stand frequency and sedentary interruption patterns. This exploratory pilot study examined whether processing identical free-living activPAL recordings using 2-second and 10-second minimum-duration settings produced different sit-to-stand, stepping, standing, and sedentary behavior outputs in chronic stroke survivors.

Methods

This study was an exploratory secondary analysis of free-living activPAL data from 12 chronic stroke survivors who were able to perform sit-to-stand transfers independently. Each raw activPAL4 recording was processed twice using PALbatch, the manufacturer-provided batch-processing software for activPAL data. In the 2-second condition, both the minimum upright period and the minimum non-upright period were set to 2 seconds. In the 10-second condition, both parameters were set to 10 seconds. The 10-second condition represented the default PALbatch setting, and all other processing settings were held constant between conditions. The primary outcome was the daily number of sit-to-stand transitions. Secondary outcomes were step count, stepping time, standing time, total sedentary time, and sedentary bout metrics. Participant-level averages across valid recording days were compared using the paired Wilcoxon signed-rank test. Secondary analyses were exploratory and were not adjusted for multiple comparisons.

Results

Daily sit-to-stand counts were higher in the 2-second condition than in the 10-second condition (52.8 ± 32.1 vs. 46.4 ± 28.2 transitions/day; mean paired difference, 6.4 transitions/day; p = 0.004). The number of sedentary bouts shorter than 30 minutes was also higher in the 2-second condition (p = 0.004). Differences in step count, stepping time, and standing time were small. Total sedentary time differed by 2.4 min/day (p = 0.035), whereas sedentary bouts lasting 30 minutes or longer showed little difference between conditions.

Conclusions

In this available-case sample, minimum-duration processing settings were primarily associated with differences in daily sit-to-stand counts and the number of short sedentary bouts. Differences in stepping-related outcomes, total sedentary time, and prolonged sedentary bout metrics were small. Because no direct observation or video-based criterion measure was available, the findings do not indicate which setting was more accurate. Instead, they show that processing settings can influence the operational definition and interpretation of activPAL-derived sit-to-stand and sedentary interruption outcomes. Researchers should report these settings explicitly and apply consistent processing rules when comparing groups, measurement occasions, or studies.

## Introduction

Sedentary behavior is generally defined as any waking behavior characterized by an energy expenditure of ≤1.5 metabolic equivalents while in a sitting, reclining, or lying posture [1]. After stroke, reduced physical activity is common, and the assessment of sedentary time and interruptions in sedentary behavior is important for understanding physical function, daily activity, and health management, including secondary prevention [2,3]. In chronic stroke survivors, objective assessment of free-living sedentary behavior is particularly relevant in both clinical and research settings because daily activity levels are often reduced [2].

The activPAL monitor (PAL Technologies, Glasgow, UK) is a thigh-worn activity monitor that has been widely used to classify posture and activity, including sitting/lying, standing, and stepping [4,5]. It has also been used to assess physical activity and sedentary behavior in community-dwelling older adults, adults in field-based studies, and populations including older adults with functional limitations [6-8]. In addition, activPAL-derived step count and stepping activity have shown acceptable validity in previous studies [9].

Sit-to-stand (STS) transitions, defined as postural transitions from a sitting or lying position to an upright position, are used as an indicator of interruptions in sedentary behavior during daily life [10]. Therefore, accurate and transparent assessment of STS counts is important when evaluating activity patterns and intervention effects in chronic stroke survivors. However, activPAL-derived STS counts may be influenced not only by the performance of the sensor itself but also by time-threshold settings used during data processing [11,12].

PALbatch is the manufacturer-provided batch-processing software used to generate posture, stepping, transition, and sedentary bout outputs from activPAL recordings. During processing, researchers can specify a minimum upright period (MUP) and a minimum non-upright period (MNUP). The MUP determines how long a standing or stepping event must last to remain as a separate upright event, whereas the MNUP specifies the corresponding minimum duration for a sitting or lying event. Events shorter than the selected duration may be combined with adjacent events during processing. Consequently, a brief standing episode between two sitting periods may be retained under a shorter setting but may not remain as a separate event under a longer setting. The default PALbatch setting for both parameters is 10 seconds [12].

Alghaeed et al. reported that a 2 s minimum sitting/upright period may minimize error in measuring sitting breaks in free-living behavior among young children when compared with direct observation [11]. In addition, a previous controlled study in healthy adults showed that MUP/MNUP settings of 1-2 s produced STS counts closer to the criterion value, whereas longer settings, particularly 10 s, substantially underestimated STS counts [12]. These findings indicate that activPAL-derived STS counts are not determined solely by device hardware but are also operationally defined by post-processing settings.

However, it remains unclear how this methodological issue affects free-living activPAL data in chronic stroke survivors. Stroke survivors may show substantial variability in walking ability, overall activity level, speed of postural transitions, and daily routines. Therefore, differences in MUP/MNUP settings may influence the interpretation of physical activity and sedentary behavior metrics in this population.

The practical question addressed in this exploratory pilot study was whether researchers could obtain different estimates of daily sit-to-stand frequency and sedentary behavior patterns when the same activPAL recordings were processed using different minimum-duration settings. We therefore processed identical free-living activPAL recordings from chronic stroke survivors using 2-second and 10-second minimum upright and non-upright periods and compared the resulting sit-to-stand, stepping, standing, and sedentary bout outputs. Because no direct observation or video-based criterion measure was available, this study was not designed to determine which setting was more accurate. Instead, it examined the extent to which processing settings could influence the reported values and interpretation of activPAL-derived outcomes.

## Materials and methods

Study design and participants

**Figure 1:**
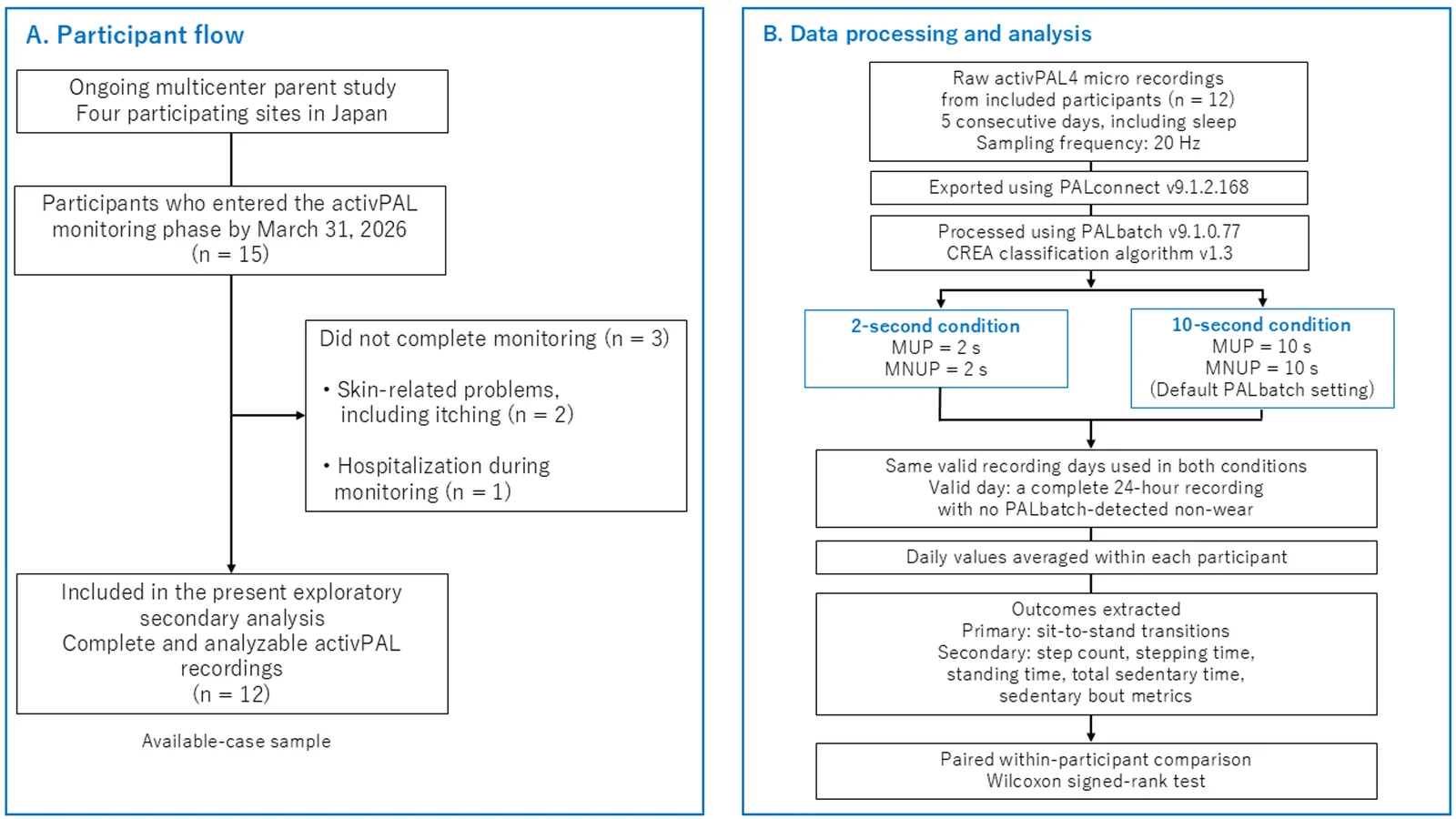
Participant flow and activPAL data processing procedure. Panel A shows the participant flow for the present exploratory secondary analysis. Panel B shows the activPAL (PAL Technologies, Glasgow, UK) data processing and analysis procedure. Identical raw activPAL recordings from the included participants were processed under the 2-second and 10-second MUP/MNUP conditions using the same valid recording days, and the resulting participant-level values were compared within participants. MUP: minimum upright period; MNUP: minimum non-upright period.

This exploratory secondary analysis used free-living activPAL recordings obtained from an ongoing multicenter study conducted at four participating sites in Japan. Figure 1 summarizes the participant flow and activPAL data processing and analysis procedure. For the present analysis, data collected and available for processing through March 31, 2026, were used. Participants were eligible for this secondary analysis if they had completed the relevant activPAL measurement procedures by the data cutoff date and had recordings that met the predefined valid-day criteria and could be processed under both conditions.

By the data cutoff date, 15 participants had entered the activPAL measurement phase relevant to the present analysis. Two participants were unable to complete activPAL monitoring because of skin-related problems, including itching, and one participant was hospitalized during the measurement period. Consequently, complete and analyzable activPAL recordings were available for 12 participants, who constituted the available-case sample for the present exploratory analysis.

Because this was an exploratory secondary analysis of data available at a defined cutoff date, no formal a priori sample size calculation was performed. The sample size was determined by the number of participants who had completed the relevant measurements by the cutoff date and whose recordings met all predefined analysis criteria.

Chronic stroke was defined as at least 6 months after stroke onset. The inclusion criteria for the parent multicenter study were as follows: (1) stroke onset at least 6 months before enrollment; (2) indoor ambulation at Functional Ambulation Categories (FAC) levels 2-5 [13]; (3) ability to perform sit-to-stand transfers independently; and (4) ability to wear the activPAL continuously in a free-living environment. The exclusion criteria for the parent study were as follows: (1) higher brain dysfunction that impaired comprehension of the study procedures, including a Mini-Mental State Examination score of 23 or lower [14], attention or memory deficits, aphasia, or unilateral neglect; (2) severe walking limitation, defined as FAC levels 0-1; and (3) cognitive, behavioral, or medical conditions judged by the attending clinician to make participation unsuitable.

For the present secondary analysis, participants were additionally required to have completed the relevant activPAL monitoring procedures by March 31, 2026, and to have at least one valid 24-hour recording day that could be processed under both the 2-second and 10-second conditions. Participants were excluded if no valid recording day remained after PALbatch non-wear screening or if their activPAL recordings could not be processed under both conditions. A valid day was defined as a complete 24-hour recording period with no non-wear time detected by PALbatch. The same valid recording days were used under both processing conditions. When more than one valid day was available, daily values were averaged separately within each processing condition to obtain one participant-level value for each outcome. Participant characteristics, including age, sex, body mass index (BMI), time since stroke onset, comfortable gait speed, and walking-aid use, were collected.

Device and attachment procedure

Physical activity and posture were recorded using an activPAL4 micro activity monitor (PAL Technologies, Glasgow, UK). The device is a thigh-worn triaxial accelerometer with a dynamic range of ±4 g. The device was initialized using the manufacturer’s software and was set to aP4 mode with a sampling frequency of 20 Hz.

In accordance with standard attachment procedures, the device was attached directly to the skin on the anterior aspect of the non-paretic thigh using medical dressing tape [7,12].

Data acquisition

Participants were instructed to wear the activPAL continuously for five consecutive days, including during sleep. Non-wear time was identified based on the non-wear detection function in PALbatch. Days with no detected non-wear time during the 24-hour recording period were considered valid days and were included in the analysis.

When participants had more than one valid day, the daily output values were averaged for each variable, and these averages were used as participant-level representative values.

Data processing

The activPAL recordings were exported using PALconnect software, version 9.1.2.168 (PAL Technologies, Glasgow, UK), the manufacturer-provided software used to retrieve recordings from the activity monitor. The recordings were subsequently processed using PALbatch software, version 9.1.0.77 (PAL Technologies), which generates posture, stepping, transition, non-wear, and sedentary bout outputs. Processing was performed using the CREA version 1.3 classification algorithm available within PALbatch. No alternative classification algorithms or external processing platforms were used.

Each participant’s raw activPAL recording was processed twice. In the 2-second condition, both the minimum upright period and the minimum non-upright period were set to 2 seconds. In the 10-second condition, both parameters were set to 10 seconds. All other PALbatch processing settings were identical between the two conditions. For readability, these conditions are hereafter referred to as the 2-second condition and the 10-second condition.

The 2-second MUP/MNUP condition was selected as a shorter-threshold condition based on previous studies showing that shorter minimum-duration settings retain more brief sitting breaks and STS-related events [11,12]. The 10-second MUP/MNUP condition was selected because it represents the default PALbatch setting and provides a practical reference condition for evaluating the influence of a longer minimum-duration threshold.

PALbatch outputs were extracted for sit-to-stand transitions, step count, stepping time, standing time, total sedentary time, and sedentary bout metrics, including the number of sedentary bouts and time spent in sedentary bouts. PALbatch-generated primary lying time was treated separately from the sedentary behavior-related outputs analyzed in the present study. Accordingly, total sedentary time and sedentary bout variables did not include primary lying time.

Outcome measures

All primary and secondary outcomes were objectively derived from the activPAL recordings using PALbatch. The primary outcome was the daily number of sit-to-stand transitions. Secondary outcomes were daily step count, stepping time, standing time, total sedentary time, and sedentary bout metrics.

Sedentary bouts were categorized as shorter than 30 minutes, 30 minutes to less than 1 hour, 1 hour to less than 2 hours, 2 hours to less than 4 hours, and 4 hours or longer. For each category, the daily number of bouts and the accumulated time spent in those bouts were calculated. No self-reported or questionnaire-based outcomes were included in the present secondary analysis.

Statistical analysis

Daily values were averaged separately within each processing condition to obtain one participant-level value for each outcome. The same valid recording days were used under the 2-second and 10-second conditions. Comparisons between conditions were therefore performed as paired within-participant comparisons.

Data are presented as mean ± standard deviation (SD). Mean paired differences were calculated as values in the 2-second condition minus those in the 10-second condition. The median of the within-participant differences was calculated in the same direction and was presented as a descriptive summary of the paired differences. The primary outcome was the daily number of sit-to-stand transitions. Secondary outcomes were step count, stepping time, standing time, total sedentary time, and sedentary bout metrics.

The normality of the paired differences was assessed using the Shapiro-Wilk test. Given the small sample size and the non-normal distribution of paired differences for several outcomes, comparisons between the two processing conditions were performed using the Wilcoxon signed-rank test. Statistical testing was not performed for outcomes for which all paired differences were zero. The primary analysis evaluated the difference in daily sit-to-stand transitions between the two conditions. Secondary outcomes were analyzed using the same paired procedure; their p-values were not adjusted for multiple comparisons and were interpreted as exploratory.

All statistical analyses were performed using R software, version 4.3.3 (R Foundation for Statistical Computing, Vienna, Austria). A two-sided p-value of <0.05 was used as the significance threshold for the primary outcome.

Ethical considerations

This study was conducted as a secondary analysis of data obtained from a study approved by the Ethics Committee of Tokushima Bunri University (approval number: R7-17). All participants received an explanation of the study and provided written informed consent.

## Results

Participant characteristics

Participant characteristics are shown in Table 1. By the data cutoff date of March 31, 2026, 15 participants had entered the activPAL monitoring phase relevant to the present analysis. Two participants did not complete monitoring because of skin-related problems, and one participant was hospitalized during the monitoring period. Consequently, 12 participants with complete and analyzable activPAL recordings were included in the final analysis. Their mean age was 70.9 ± 10.0 years, and their mean body mass index (BMI) was 24.8 ± 6.3 kg/m². The mean time since stroke onset was 120.5 ± 74.7 months, and the mean comfortable gait speed was 0.65 ± 0.19 m/s. Six participants were male, and six were female. Although individuals with Functional Ambulation Categories levels 2-5 were eligible for the parent study, all 12 participants included in the present analysis had levels 3-5. Regarding walking-aid use, two participants walked without an aid, nine used a cane, and one used a wheeled walker.

**Table 1:** Participant characteristics Continuous variables are presented as mean ± standard deviation, and categorical variables are presented as n (%).

Characteristic	Value
Age, years	70.9 ± 10.0
Sex, Male	6 (50.0)
Sex, Female	6 (50.0)
BMI, kg/m²	24.8 ± 6.3
Time since stroke onset, months	120.5 ± 74.7
Comfortable gait speed, m/s	0.65 ± 0.19
Walking aid	
No aid	2 (16.7)
Cane	9 (75.0)
Wheeled walker	1 (8.3)

Comparison of activPAL-derived outcomes between the 2-second and 10-second conditions

The comparison of activPAL-derived outcomes between the 2-second and 10-second conditions is shown in Table 2. The number of sit-to-stand transitions was significantly higher in the 2-second condition than in the 10-second condition. Sit-to-stand transitions were 52.8 ± 32.1 transitions/day in the 2-second condition and 46.4 ± 28.2 transitions/day in the 10-second condition. The mean paired difference was 6.4 transitions/day, and the median paired difference was 6.3 transitions/day (p = 0.004).

**Table 2:** Comparison of activPAL-derived outcomes between the MUP/MNUP 2 s and 10 s conditions Values are based on participant-level averages (n = 12). When multiple valid days were available, the mean across valid days was used as the participant-level representative value. Mean differences and median paired differences were calculated as values in the MUP/MNUP 2-second condition minus those in the MUP/MNUP 10-second condition. Median paired differences are presented as descriptive summaries of the within-participant differences. The p-values were calculated using the Wilcoxon signed-rank test based on paired within-participant differences. A dash (—) indicates variables for which all paired differences were zero and statistical testing was therefore not performed. The 2-second and 10-second conditions refer to MUP = MNUP = 2 s and MUP = MNUP = 10 s, respectively. P-values for secondary outcomes were not adjusted for multiple comparisons and should be interpreted as exploratory. MUP: minimum upright period; MNUP: minimum non-upright period; SD: standard deviation.

Outcome	MUP/MNUP 2 s condition, mean ± SD	MUP/MNUP 10 s condition, mean ± SD	Mean difference (2 s − 10 s)	Median paired difference (2 s − 10 s)	p-value
Sit-to-stand transitions, n/day	52.8 ± 32.1	46.4 ± 28.2	6.4	6.3	0.004
Step count, steps/day	2484.8 ± 2650.0	2486.2 ± 2651.5	-1.4	0.0	0.281
Stepping time (min/day)	37.8 ± 39.0	37.9 ± 39.1	0.0	0.0	0.281
Standing time (min/day)	167.1 ± 133.5	168.7 ± 135.8	-1.6	0.0	1.000
Total sedentary time (min/day)	792.7 ± 185.5	790.4 ± 185.1	2.4	0.3	0.035
Sedentary bouts <30 min (n/day)	44.6 ± 31.0	38.3 ± 27.3	6.4	6.3	0.004
Sedentary bouts 30-60 min (n/day)	3.9 ± 2.1	3.8 ± 2.0	0.1	0.0	0.500
Sedentary bouts 1-2 h (n/day)	1.6 ± 1.4	1.7 ± 1.3	-0.1	0.0	1.000
Sedentary bouts 2-4 h (n/day)	0.8 ± 0.8	0.8 ± 0.8	0.0	0.0	1.000
Sedentary bouts ≥4 h (n/day)	0.5 ± 0.6	0.5 ± 0.6	0.0	0.0	—
Time in sedentary bouts <30 min (min/day)	212.4 ± 129.2	205.3 ± 128.8	7.1	0.2	0.055
Time in sedentary bouts 30-60 min (min/day)	162.5 ± 91.6	159.5 ± 88.9	3.0	0.0	0.625
Time in sedentary bouts 1-2 h (min/day)	137.3 ± 127.1	141.2 ± 121.3	-3.9	0.0	0.625
Time in sedentary bouts 2-4 h (min/day)	140.3 ± 139.7	144.2 ± 143.1	-3.8	0.0	1.000
Time in sedentary bouts ≥4 h (min/day)	140.2 ± 184.4	140.2 ± 184.4	0.0	0.0	—

In contrast, stepping-related outcomes showed minimal mean differences between the two conditions. Step count was 2484.8 ± 2650.0 steps/day in the 2-second condition and 2486.2 ± 2651.5 steps/day in the 10-second condition, with a mean difference of -1.4 steps/day (p = 0.281). Stepping time was 37.8 ± 39.0 min/day in the 2-second condition and 37.9 ± 39.1 min/day in the 10-second condition, with a mean difference of 0.0 min/day (p = 0.281). Standing time was 167.1 ± 133.5 min/day in the 2-second condition and 168.7 ± 135.8 min/day in the 10-second condition, with a mean difference of -1.6 min/day (p = 1.000).

Total sedentary time was 792.7 ± 185.5 min/day in the 2-second condition and 790.4 ± 185.1 min/day in the 10-second condition. The mean paired difference was 2.4 min/day (p = 0.035); however, the absolute difference was small.

In the sedentary bout analysis, the difference between the two MUP/MNUP settings was mainly observed in short sedentary bouts lasting less than 30 min. The number of sedentary bouts lasting less than 30 min was higher in the 2-second condition than in the 10-second condition (44.6 ± 31.0 vs. 38.3 ± 27.3 bouts/day; mean paired difference, 6.4 bouts/day; p = 0.004). No clear between-condition differences were observed in the number of sedentary bouts lasting 30-60 min, 1-2 h, or 2-4 h. For sedentary bouts lasting 4 h or longer, all paired differences were zero, and statistical testing was therefore not performed.

Time spent in sedentary bouts lasting less than 30 min was 212.4 ± 129.2 min/day in the 2-second condition and 205.3 ± 128.8 min/day in the 10-second condition. Although the mean value was 7.1 min/day higher in the 2-second condition, no clear between-condition difference was observed (p = 0.055). No clear between-condition differences were observed in time spent in sedentary bouts lasting 30-60 min, 1-2 h, or 2-4 h. For time spent in sedentary bouts lasting 4 h or longer, all paired differences were zero, and statistical testing was therefore not performed.

## Discussion

In this exploratory pilot study, we reprocessed the same free-living activPAL data from 12 chronic stroke survivors using two different PALbatch MUP/MNUP settings: the 2-second condition and the 10-second condition. The primary finding was that the daily number of sit-to-stand transitions differed between the two processing conditions. In the exploratory analyses of secondary outcomes, a difference was also observed in the number of sedentary bouts shorter than 30 min. In contrast, step count, stepping time, standing time, and prolonged sedentary bout metrics showed minimal differences between the two conditions.

The clearest processing-related difference was observed in the daily number of sit-to-stand transitions. On average, the 2-second condition produced 6.4 more transitions per day than the 10-second condition. This difference could reflect the retention of brief upright events that did not remain as separate events under the longer minimum-duration setting. However, no synchronized direct observation or video-based criterion measure was available. It is therefore not possible to determine whether the additional events retained under the 2-second condition represented genuine sit-to-stand transitions, transient postural events of uncertain clinical relevance, or a combination of both. Accordingly, the finding should be interpreted as a processing-related difference in output rather than evidence that the 2-second condition provided greater detection accuracy. The results indicate that activPAL-derived STS counts are not determined solely by sensor-recorded events but are also operationally defined by the MUP/MNUP settings applied during post-processing.

In contrast to sit-to-stand transitions, stepping-related outcomes showed minimal differences between the two conditions. Step count, stepping time, and standing time showed little difference between the 2-second and 10-second conditions. This finding indicates that changing MUP/MNUP settings may have limited influence on the overall amount of walking or upright time, while having a greater effect on event-based outcomes such as sit-to-stand transitions. In practical terms, MUP/MNUP settings may matter more when the research question focuses on how often a person changes posture than when it focuses on the total volume of stepping or standing.

The analysis of sedentary bouts also supports this interpretation. The difference between the two MUP/MNUP settings was mainly observed in the number of sedentary bouts shorter than 30 min. In contrast, sedentary bouts lasting 30 min or longer, including prolonged bouts of 2 h or longer, showed little difference between conditions. These results suggest that MUP/MNUP settings mainly influence the detection and segmentation of short sedentary bouts and brief interruptions, rather than substantially altering the classification of longer sedentary periods.

Although the comparison of total sedentary time yielded a p-value below 0.05, the mean difference was only 2.4 min/day. Given the exploratory analysis of multiple secondary outcomes and the small absolute difference, this finding should be interpreted cautiously and is unlikely to represent a meaningful difference in total sedentary exposure in this sample. The effect of MUP/MNUP settings appears to be concentrated more on sit-to-stand transitions and short sedentary bout metrics than on total sedentary time.

These findings have practical implications for studies using activPAL-derived STS counts in chronic stroke survivors. STS counts are often interpreted as an indicator of interruptions in sedentary behavior and daily postural transitions. However, the present results indicate that these counts can vary depending on MUP/MNUP settings, even when the same raw activPAL data are used. Therefore, studies that report STS counts should clearly describe the MUP/MNUP settings used during PALbatch processing. In addition, caution is needed when comparing STS counts across studies that use different post-processing settings.

The magnitude of the observed difference should also be interpreted in relation to the daily frequency of STS transitions in the present sample. The daily STS counts were 52.8 transitions/day in the 2-second condition and 46.4 transitions/day in the 10-second condition. These values were higher than those reported during stroke rehabilitation, including 25.0 ± 17.2 transitions/day in the overall monitored group and 34.1 ± 12.4 transitions/day among participants who were independent in STS performance [15]. However, they were lower than the 71 ± 25 transitions/day reported for healthy older adults living in the community [16]. In addition, STS transitions have been used as objective indicators of sedentary behavior patterns in ambulatory people with stroke [17]. However, these comparisons should be interpreted cautiously because the measurement environments, participant characteristics, monitoring durations, and processing methods differed across studies. Therefore, the present findings may not represent the full range of possible processing-related differences in individuals with higher daily transition frequency. In more active individuals, or in those with more frequent brief upright or non-upright events, the absolute difference between shorter and longer MUP/MNUP settings could be larger.

This issue is particularly relevant when STS counts are used as outcomes in intervention studies or longitudinal monitoring. A study in community-dwelling older adults reported a minimum detectable change of 20.1 STS transitions/day for free-living STS volume [18]. Although this value cannot be directly applied as a threshold for the present sample because it was derived from a different population and processing algorithm, it provides contextual information for interpreting the magnitude of the observed processing-related difference. Together with evidence that activPAL processing choices can affect posture and activity outputs, particularly transition and sedentary bout metrics [11,12,19], the present findings suggest that activPAL-derived STS counts obtained using different PALbatch MUP/MNUP settings may not be directly comparable. Therefore, intervention and longitudinal studies should use identical MUP/MNUP settings across baseline and follow-up assessments and should interpret small changes in STS counts with caution.

Although the present study cannot determine which MUP/MNUP setting is closer to the true number of STS events, the finding that the 2-second condition yielded higher STS counts is consistent with previous studies suggesting that shorter minimum period settings may be more sensitive to brief sitting breaks or STS transitions [11,12]. Alghaeed et al. reported that a 2 s minimum sitting/upright period may minimize error in measuring sitting breaks when compared with direct observation in young children [11]. In addition, a previous controlled study in healthy young men showed that shorter MUP/MNUP settings, particularly 1-2 s, produced STS counts closer to the criterion value, whereas the 10 s setting underestimated brief STS transitions [12]. Therefore, shorter MUP/MNUP settings may be particularly relevant when the research focus is on brief STS transitions or sedentary interruption metrics. However, this interpretation should be confirmed in chronic stroke survivors using direct observation or video-based reference measures.

Overall, the results suggest that PALbatch MUP/MNUP settings should be considered when interpreting activPAL-derived STS counts and sedentary interruption metrics in chronic stroke survivors. For outcomes related to overall stepping or prolonged sedentary time, the influence of these settings may be relatively small. For event-based outcomes, especially STS counts and short sedentary bouts, prespecification, consistent application, and explicit reporting of MUP/MNUP settings are important.

An important strength of this study was the paired reprocessing of identical raw recordings. Each participant’s recording was analyzed under both processing conditions while all other settings were held constant. This design minimized the influence of between-day behavioral variability and allowed the observed differences to be attributed specifically to the processing rules applied to the same recordings.

Limitations

This study has several limitations. First, it was an exploratory secondary analysis based on an available-case sample of 12 chronic stroke survivors, and no formal a priori sample size calculation was performed. The present analysis included participants with complete and analyzable activPAL recordings available by the data cutoff date within an ongoing multicenter parent study. This selection process may have introduced selection bias, and the analyzed sample may not fully represent the participant characteristics or the magnitude of processing-related differences in the full parent-study cohort. The small sample size also limited statistical precision, subgroup analyses, and generalizability.

Second, the eligibility criteria required participants to be able to perform sit-to-stand transfers independently and to walk indoors at Functional Ambulation Categories levels 2-5. Moreover, all participants included in the present analysis had Functional Ambulation Categories levels 3-5. The findings may therefore not apply to stroke survivors who require substantial physical assistance, have more severe mobility impairment, or exhibit markedly different daily activity patterns.

Third, no direct observation, synchronized video recording, or behavioral log was available as a criterion measure. Consequently, the study could not determine whether the 2-second or 10-second condition was closer to the true number of sit-to-stand events and should not be interpreted as a validation study. The additional events retained under the 2-second condition may have represented genuine brief sit-to-stand transitions, transient postural events of uncertain clinical relevance, or a combination of both.

Fourth, the present study compared processing outputs generated from the same recordings at a single measurement period. Therefore, it could not determine whether the magnitude of processing-related differences remains consistent over time or how the settings affect estimates of change in intervention or longitudinal studies.

Fifth, the small sample size prevented examination of whether gait speed, transition speed, walking-aid use, stroke characteristics, overall activity level, or other participant factors modified the magnitude of the processing-related differences.

Sixth, multiple secondary outcomes were analyzed exploratorily without adjustment for multiple comparisons. Findings concerning total sedentary time and individual sedentary bout categories should therefore be interpreted cautiously.

Future directions

Future studies should evaluate minimum-duration processing settings in larger and more diverse samples of stroke survivors. Direct observation or synchronized video should be used as a criterion measure to determine whether events retained under shorter processing settings represent genuine sit-to-stand transitions. Further studies should also examine whether mobility level, transition speed, and daily activity patterns modify processing-related differences and whether these settings influence the detection of behavioral changes in longitudinal and intervention studies.

## Conclusions

In this exploratory pilot study using free-living activPAL data from chronic stroke survivors, the daily number of sit-to-stand transitions differed between the 2-second and 10-second PALbatch MUP/MNUP conditions. In exploratory analyses of secondary outcomes, a difference was also observed in the number of sedentary bouts shorter than 30 min, whereas step count, stepping time, standing time, and prolonged sedentary bout metrics showed minimal differences between conditions. These findings demonstrate that, even when identical raw activPAL recordings are used, the calculated number of sit-to-stand transitions may vary according to the MUP/MNUP settings applied during post-processing. Because no direct observation or synchronized video-based criterion measure was available, the findings should not be interpreted as evidence that either setting was more accurate. Rather, the results indicate that MUP/MNUP settings contribute to the operational definition of activPAL-derived sit-to-stand transition counts. Therefore, when sit-to-stand counts or sedentary interruption metrics are used in chronic stroke research, MUP/MNUP settings should be prespecified, applied consistently, and clearly reported. Larger studies using criterion-based observations are needed to clarify how these settings affect sit-to-stand detection in free-living environments.
